# Capecitabine in adjuvant radiochemotherapy for gastric adenocarcinoma

**DOI:** 10.2478/raon-2013-0065

**Published:** 2014-04-25

**Authors:** Irena Oblak, Marija Skoblar Vidmar, Franc Anderluh, Vaneja Velenik, Ana Jeromen, Jasna But Hadzic

**Affiliations:** Department of Radiotherapy, Institute of Oncology Ljubljana, Ljubljana, Slovenia

**Keywords:** gastric cancer, capecitabine, adjuvant therapy, radiochemotherapy, survival, toxicity

## Abstract

**Background:**

In patients with non-metastatic gastric cancer surgery still remains the treatment of choice. Postoperative radiochemotherapy with 5-fluorouracil and leucovorin significantly improves the treatment outcome. The oral fluoropyrimidines, such as capecitabine, mimic continuous 5-fluorouracil infusion, are at least as effective as 5-fluorouracil, and such treatment is more comfortable for the patients.

**Patients and methods.:**

In the period from October 2006 to December 2009, 101 patients with gastric cancer in stages Ib–IIIc were treated with postoperative chemoradiation with capecitabine. Distal subtotal resection of the stomach was performed in 46.3%, total resection in 50.5% and multivisceral resection in 3.2% of patients. The main endpoints of this study were loco-regional control (LRC), disease-free survival (DFS), disease-specific survival (DSS) and overall survival (OS). The rates of acute side-effects were also estimated.

**Results:**

Seventy-seven percent of patients completed the treatment according to the protocol. The median follow-up time of all patients was 3.9 years (range: 0.4–6.3 years) and in survivors it was 4.7 years (range: 3.2–6.3 years). No death occurred due to the therapy. Acute toxicity, such as nausea and vomiting, stomatitis, diarrhoea, hand-foot syndrome and infections of grade 3 or 4, occurred in 5%, 1%, 2%, 8.9% and 18.8% of patients, respectively. On the close-out date 63.4% patients were still alive and with no signs of the disease. The 4-years follow-up survey showed that LRC, DFS, DSS and OS were 95.5%, 69.2%, 70.7%, and 66.2%, respectively. Higher pN-stage and splenectomy were found to be independent prognostic factors for all four types of survival and perineural invasion and lower treatment intensity for DFS, DSS and OS.

**Conclusions:**

Postoperative radiochemotherapy with capecitabine is feasible, with low toxicity and the results of such treatment are good.

## Introduction

Although the incidence of gastric cancer has declined in recent years it is still one of the most common causes of cancer death.^1^,^2^ Complete removal of tumour masses with regional lymph nodes (so called R0 surgical resection) represents the treatment of choice in patients with non-metastatic gastric cancer.^2^ The standard recommendations for lymphadenectomy are at least D1 resection and the removal of a minimum of 15 lymph nodes.^3^,^4^ Although the R0 surgical resection is performed, patients’ survival remain unsatisfactory. During the past few decades, the principle of combined modality treatment has been developed and applied in practice for various solid tumours with gastric cancer not being an exception. One of the landmark studies in adjuvant trials was the Intergroup Study INT-0116, in which a significant improvement in survival with the use of 5-fluorouracil (5-FU) based radiochemotherapy after surgery was reported.^5^ After this study radiochemotherapy was established as routine adjuvant treatment in the USA, as well as in several European countries. In the Institute of Oncology Ljubljana, the program of combined postoperative treatment of non-metastatic gastric carcinoma with radiochemotherapy was introduced into clinical practice in 2001. The results of that regimen were analysed and presented.^6^,^7^

It is well known that capecitabine is an oral 5-FU prodrug and we assume that it can replace standard chemotherapy. It has been proven that capecitabine mimics continuous 5-FU infusion^8^,^9^ and is at least as effective as 5-FU10,11, but with less side effects.^9^ In addition, treatment with the oral fluoropyrimidines, such as capecitabine, is more comfortable for patients because these drugs can be taken at home, without any invasive procedures (such as application of parenteral infusion of chemotherapy). We speculate that radio sensibilisation with capecitabine could be more effective than with 5-FU, because 5-FU is given only on the first four and last three days of radiotherapy, whereas capecitabine is given through the whole course of the radiotherapy. The aim of this study was to evaluate the efficacy and toxicity of adjuvant radiochemotherapy with capecitabine.

## Patients and methods

### Patients

In the period from October 2006 to December 2009, 101 patients (66 males and 35 females, aged 26–78 years, mean age 58.9 years) were treated for non-metastatic adenocarcinoma of non-cardial gastric cancer with postoperative concomitant chemoradiation with capecitabine at the Institute of Oncology, Ljubljana, Slovenia. All patients had locally and/or regionally advanced disease without distant metastases (stages Ib–IIIc).^12^ Before the start of the treatment 62 (61.4%) patients suffered from epigastrial pain and 25 (24.8%) patients complained of early satiety. Anaemia was found in 28 (27.7%) patients, melena in 17 (16.8%) patients and weight loss in 57 (56.4%) patients.

### Surgical treatment

Of the 101 patients, 83 (82.1%) were operated on in two major surgical centres in Slovenia (at the University Medical Centre Ljubljana or Maribor) and the remaining 18 (17.1%) patients in one of five Slovenian regional hospitals. Distal subtotal resection of the stomach was performed in 47 (46.5%) patients, total resection of the stomach in 51 (50.5%) patients and multivisceral resection in three (3%) patients, respectively. Radical resection (R0) of the stomach was performed in 97 (96%) patients and the remaining four (4%) patients underwent non-radical surgery (R1) with no possibility of reoperation. At least 15 lymph nodes were removed and histologically examined in 70 (69.3%) patients and less than 15 lymph nodes were examined in 31 (30.7%) patients.

### Tumour characteristics

Primary tumours originated in the antrum in 49 (48.5%) patients, in the corpus in 38 (37.6%) patients, in the lesser curvature in 10 (9.9%) and in the greater curvature in four (4%) patients. In 47 (46.5%) patients, the tumour was staged as pT3 or pT4, and 80 (79.2%) patients had N+ disease. Sixty-five (64.4%) tumours were poorly differentiated (G3) (Table 1).

### Investigations before and during therapy

After surgery, all patients with the disease in pathological stage Ib or more were presented to a multi-disciplinary advisory team, consisting of a surgeon, radiation oncologist and medical oncologist, in order to assess the prospects of eventual adjuvant treatment. Patients had to fulfil the following criteria: histologically confirmed adenocarcionoma of the stomach, cancer removed with R0 or R1 re-section, age greater than 18 and below 80 years, a performance status of 1 or lower according to the World Health Organization (WHO), adequate function of major organs (including cardiac, bone marrow, renal and hepatic function), no difficulty in swallowing tablets and adequate collaboration during treatment. All patients underwent a general clinical examination and blood counts. The investigations, such as X-ray, ultrasound (US), and/or computer tomography (CT) of the thorax or abdomen, performed before surgery to rule out meta-static disease, were repeated only in the patients in whom the progression of the disease was clinically suspected. During the therapy, the patients were clinically examined and referred to haematology and biochemistry blood tests once a week. The therapy-related local and systemic toxicity was assessed according to the National Cancer Institute Common Toxicity Criteria (NCI-CTC) version 2.0.^13^ The performance status of patients was determined and their body weight was measured on a weekly basis.

### Postoperative radiochemotherapy

Adjuvant treatment was initiated within 6–8 weeks after surgery and consisted of concomitantly applied chemo- and radiotherapy. Chemotherapy started with peroral capecitabine 1250 mg/m^2^ twice daily (bid) on days 1–14, with a one week break. Concurrently with irradiation, continuous capecitabine 825 mg/m^2^ bid was administered, without weekend breaks. After the completion of radiotherapy with two weeks break, the patients received three more cycles of capecitabine 1250 mg/m^2^ bid on days 1–14, with a one week break between each cycle.

Patients were irradiated on linear accelerator with 15 MV photon beams for five days per week, at a daily dose of 1.8 Gy. Radiotherapy planning was performed using simulator with CT option and 3-D treatment planning computer software. The total irradiation dose was 45 Gy delivered in five weeks. The clinical target volume (CTV) was defined using preoperative CT, endoscopic findings, surgical clips and findings during operation. In CTV tumour bed, anastomosis site, duodenal stump, remnant stomach and regional lymph nodes were enclosed, and it extended 2.5 cm beyond the proximal and distal margins of resection. The irradiation dose was specified according to the International Commission on Radiation Units (ICRU) recommendations.

In case of severe therapy-related toxicity, irradiation and/or chemotherapy doses were modified and adapted to the patient’s physical condition or laboratory tests. When necessary, chemotherapy application was delayed or radiotherapy was temporarily interrupted or terminated.

### Statistics

Statistical analysis was performed using personal computer and software statistical package SPSS, version 15 (SPSS Inc., USA). The main endpoints of this study were as follows: locoregional control (LRC; the event was local and/or regional recurrence), disease-free survival (DFS; the event was local, regional or systemic recurrence), disease-specific survival (DSS; the event was death due to gastric adenocarcinoma) and overall survival (OS; the event was death from any cause).

The survival of patients was computed from the date of the surgery to January 1st, 2013 (close-out date). Survival probability was calculated using the Kaplan-Meier estimate^14^, and log rank test^15^ was used to evaluate the differences between individual groups of patients. Independent prognostic values of variables that appeared as statistically significant on univariate analysis were tested by multivariate Cox regression analysis model.^16^ Two-sided tests were used and differences of p < 0.05 were considered as statistically significant.

## Results

### Toxicity of adjuvant radiochemotherapy

Postoperative chemotherapy started 2.6–11.2 weeks after surgery (median 6 weeks). Total postoperative treatment time ranged from 4.3 to 29.3 weeks (median 17.1 weeks), whereas the median duration of the radiotherapy part of the protocol was 4.7 weeks. Seventy-seven percent of patients completed the treatment according to the protocol. Ninety-seven (96%) patients reached the total radiation dose of 45 Gy, whereas in four patients (4%) the total dose was lower (two patients received 9 Gy, one 32.4 Gy and one 34.2 Gy, respectively). The other 19 (18.8%) patients who did not complete the treatment according to the protocol did not receive all cycles of chemotherapy (one patient received two cycles, 7 patients three cycles and 11 patients four cycles). No death occurred due to the therapy. Acute toxicity, such as nausea and vomiting, stomatitis, diarrhoea, hand-foot syndrome and infections of grade 3 or 4, occurred in 5%, 1%, 2%, 8.9% and 18.8% of patients, respectively (Table 2). Despite intensive nutritional support, only in two patients an increase of body weight was recorded during the therapy. Forty (39.6%) patients maintained constant weight, whereas the remaining 59 (58.4%) patients lost their weight compared to the weight they had at the beginning of treatment. The body weight loss was 1–17 kg (median 5 kg).

### Outcome

**FIGURE 1.**
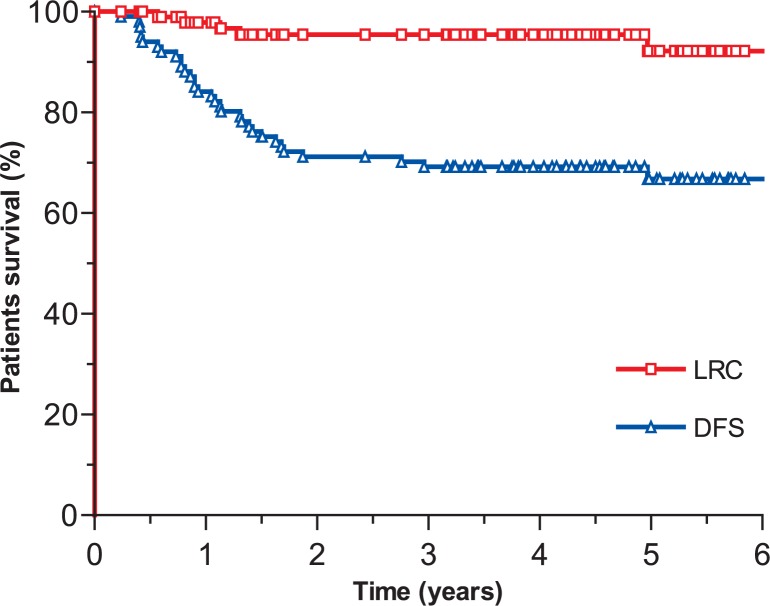
Locoregional control (LRC) and disease-free survival (DFS).

**FIGURE 2.**
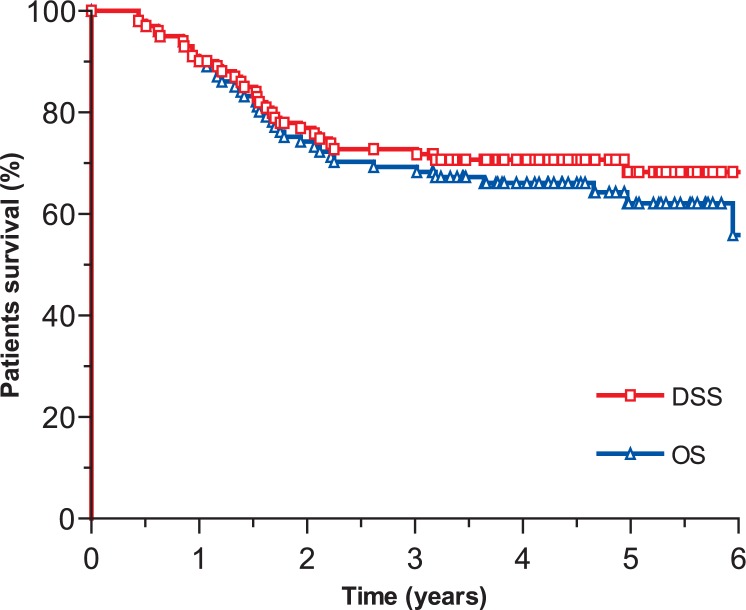
Disease-specific survival (DSS) and overall survival (OS).

The median follow-up time of all 101 patients was 3.9 years (range: 0.4–6.3 years), whereas in survivors it was 4.7 years (range: 3.2–6.3 years). On the close-out date, 64 (63.4%) patients were still alive, all of them being with no signs of the disease. Thirty (29.6%) patients died from gastric carcinoma, five (5%) patients died from other causes and in two (2%) patients the cause of death could not be determined. After adjuvant radiochemotherapy, recurrence was observed in 32 (31.7%) patients. Local and/or regional recurrence developed in five (5%) patients after a median period of time of 1.1 year (range: 0.6–1.3 years). Systemic disease alone developed in 27 (26.7%) patients in the median period of time of 0.9 year (range: 0.2–3 years). The 4-years follow-up survey showed that LRC, DFS, DSS and OS were 95.5%, 69.2%, 70.7%, and 66.2%, respectively (Figures 1 and 2).

### Prognostic factors

On a univariate analysis of survival, the patients with pN3-stage, low pretreatment haemoglobin (Hb) concentration ≤ 120 g/l and age above 70 years had lower locoregional control and survival in comparison to their counterparts in all four survival endpoints. In addition, poorer treatment outcome correlated also with locally advanced disease (pT3-4), overall disease stage III, perineural invasion, lower treatment intensity (patients who did not complete the treatment according to the protocol and patients who started with adjuvant radiochemotherapy in more than 6 weeks after surgery), low Hb (< 110 g/l) during radiochemotherapy and with splenectomy performed. The multivariate analysis identified the more advanced pN-stage and splenectomy as independent prognostic factors for all four types of survival. Independent prognostic factors for DFS, DSS and OS were perineural invasion and lower treatment intensity (Table 3).

## Discussion

For gastric cancer, complete resection is the only curative therapy. Unfortunately, more than 50% of patients are diagnosed with unresectable disease.^17^ In patients who underwent radical resection the 5-years survival rate is lower than 30%^18^ with the rate of locoregional recurrence up to 50–80%.^19^,^20^ For this reason, patients with gastric adenocarcinoma in many countries receive postoperative radiochemotherapy with 5-FU in combination with leucovorin (LV), because it has been proven that it significantly improves the survival of these patients.^4^,^5^,^20^–^25^

An updated analysis of INT 0116 study of 556 patients with resectable adenocarcinoma of the stomach or gastroesophageal junction showed that postoperative radiochemotherapy with 5-FU and LV improves 5-years overall survival (40% vs. 22%) and local recurrence rate (19% vs. 22%) compared with surgery alone.^22^ However, this study was criticized by some due to poorly performed surgery. In 54% of patients only D0 lymphadenectomy instead of the recommended D2 lymphadenectomy was performed. When comparing these results with those of Kim′s study in which all patients underwent D2 lymphadenectomy, the 5-years survival rate was 57.1% in the radiochemotherapy arm and 51% in the surgery only group.^21^ In the study of Park *et al.* in all patients D2 lymphadenectomy was performed and the results were similar with the 5-years survival rate of 60%.^20^ As the same adjuvant regimen was used in both studies, it seems that the reason for better outcome in both studies is a more extensive lymphadenectomy. This thinking seems to be confirmed also in the Dutch study, where there was only a little benefit from adjuvant radiochemotherapy in patients with D2 lymphadenectomy in comparison to those with D1 limphadenectomy.^23^ The ARTIST trial, in which patients after radical resection of gastric cancer were randomized in the group treated with capecitabine and cisplatin and in the group treated with the same regiment of chemotherapy plus radiotherapy, reported that in the group treated with postoperative radiochemotherapy a statistical trend towards better DFS was observed. The benefit of postoperative radiochemotherapy was even higher in the subgroup of patients with positive lymph nodes.^24^ In a similarly designed trial, Chinese experts concluded that adjuvant radiochemotherapy with intensity-modulated radiotherapy (IMRT) significantly improves 5-years DFS in comparison with postoperative chemotherapy in the whole patient population, not only in those with positive lymph nodes.^25^ In the above two trials and in the trial of Yu *et al.*^26^ it has been noticed that adjuvant radio-chemotherapy in the patients with gastric cancer gives higher benefit in more advanced stages of the disease (but without distant metastases). The survival benefit noted in patients who were treated with radiochemotherapy was entirely due to an improvement in local control with less effect on distant metastases, which suggests that the chemotherapy with 5-FU and LV is producing its effect through radiosenzitisation.^4^

In our study the surgeons were obliged to follow the protocol and to perform routinely at least D1 lymphadenectomy. Fifteen or more lymph nodes were removed and histologically examined in 70 (69.3%) patients and less than 15 lymph nodes were examined in 31 (30.7%) patients. In only 4 patients the resection was estimated as R1 and reoperation in the opinion of surgeons was not possible.

In our previous report, long term results of adjuvant radiochemotherapy with 5-FU and LV in patients with gastric cancer were analyzed with the 5-years LRC, DFS, DSS and OS of 81%, 48.3%, 50.4% and 48.4%, respectively.^7^ In the reports of Macdonald *et al.*^5^, Kim *et al.*^21^ and ours, chemotherapy with 5-FU and LV was given concomitantly during radiotherapy only in the first four and last three days. In our current study with administration of capecitabine on each day of radiotherapy, we hoped for better radiosensitization effects and a better treatment outcome. Jansen *et al.* designed the phase I-II study with postoperative radiochemotherapy with capecitabine and they reported that such treatment is feasible with low toxicity.^26^ The estimated 5-years follow-up survey in our study showed that LRC, DFS, DSS and OS were 92.2%, 66.8%, 68.3%, and 62.1%, respectively. These results were better in comparison to other data^5^,^20^–^23^ which can be attributed to the fact that chemotherapy with capecitabine instead of 5-FU and LV was used. On the other hand, these good results could be due to careful selection of patients because we excluded all the patients with tumours located in the cardia (known to have a worse treatment outcome), patients who were not able to take capecitabine (difficulty in swallowing tablets or unable to comply) and patients with significant co-morbidities.

The other criticism of the American intergroup study was referred to the high percentage of patients (36%) who did not conclude the therapy according to the protocol.^5^,^22^ In Kim′s study, 24.8% of enrolled patients with radiochemotharapy did not complete the treatment as planned.^21^ They also reported that, due to chemotherapy related toxicity, the dose of the drugs had to be reduced in 48.9%, whereas in 24.5% of patients the application of chemotherapeutics had to be delayed. All above mentioned authors nevertheless believe that the INT 0116 protocol is safe and acceptable for clinical use and we also support this opinion.^7^ In our study, in which radiochemotherapy with capecitabine was used, only 19 (18.8%) patients did not complete the treatment according to the protocol which is more favourable than the other authors′ reports for radiochemotherapy with 5-FU and LV.^5^,^20^ In our previous study with radiochemotherapy with 5-FU and LV, we obtained the same results with only 18% of patients who were not able to complete the treatment. We believe that in our case, this favourable experience may be due to the fact that we insisted on extensive advising of our patients on all potential side-effects of chemo- and radiotherapy. Furthermore, all of the patients received intensive supportive care, including intensive nutritional support.

If we look at acute toxicity more precisely, we can conclude that it is low and feasible. Nausea and vomiting, stomatitis, diarrhoea, hand-foot syndrome and infections of grade 3 or 4 in our current study occurred in 5%, 1%, 2%, 8.9% and 18.8% of patients. In our previous study, where radio-chemotherapy with 5-FU and LV was used, nausea and vomiting, stomatitis, diarrhoea and infections of grade 3 or 4, occurred in 18.7%, 26%, 8.9% and 12.2% of patients, respectively.^6^ In Macdonald′s study the gastrointestinal type of toxic effects of grade 3 or more occurred in 33% of patients, infection in 6% and there were three deaths due to the therapy.^5^ In our study we did not have any death related to the treatment. In the study of Park *et al.* nausea, stomatitis and diarrhoea of grade 3 or more occurred in 12%, 15% and in 11% of patients, respectively.^20^ In the study of Lee *et al.*^24^ and Zhu *et al.*^25^ the toxicity profile was a bit better with nausea in 12.3 and 2.7% and vomiting in 3.1% and 1.6% of patients. In Zhu’s study diarrhoea was present in 1.6% and in Lee’s study hand-foot syndrome was present in 3.1% of patients. Jansen *et al.* who used capecitabine in doses of 650 mg/m^2^, 800 mg/m^2^, 900 mg/m^2^ and 1000 mg/m^2^ with radiotherapy, did not notice any grade 3 or more side effects such as nausea, vomiting, diarrhoea and hand-foot syndrome.^27^

From our analysis of prognostic factors, we may conclude that the patients with more advanced pN-stage and/or underwent splenectomy and/ or had perineural invasion and/or lower treatment intensity have lower survival in comparison to their counterparts. More advanced pN-stage is considered to be well established negative prognostic factor for patients with gastric cancer and is also usually mentioned as such in pertinent literature.^28^–^32^ Some researchers noted that splenectomy has an adverse effect on patients’ survival.^5^,^33^ This has been shown in our study, too. Splenectomy is recommended only for patients with direct tumour invasion in the spleen or in the advanced gastric cancer located in the proximal part of the stomach, when there is evidence of macroscopic invasion into serosal surface and with regional lymph node metastasis.^34^,^35^ Otherwise its negative effect on postoperative morbidity and mortality is too strong and it prevails over treatment benefit. Perineural invasion has already been established as an important negative prognostic factor in our previous study of adjuvant radiochemotherapy with 5-FU and leucovorin.^6^ It is well known that the intensity of therapy can have an influence on treatment outcome in many neoplasms.^6^,^7^,^31^,^36^

In conclusion, we emphasize that multidisciplinary approach is mandatory for taking the decision about the treatment of patients with gastric cancer. Adjuvant radiochemotherapy with capecitabine is acceptable for clinical use, because it gives encouraging results regarding patients’ survival and low toxicity. However, because the local control with the existing treatment is excellent, in our opinion, to improve the outcome for these patients and reduce the rate of distant metastases, the new generation of systemic therapy combinations which could be used with the irradiation is needed.

## Figures and Tables

**TABLE 1.** Pathohistologic characteristics of tumours

**Characteristic**		**No. of patients**	**%**
pT – stage	1a	1	1
	1b	5	5
	2	48	47.5
	3	40	39.5
	4a	2	2
	4b	5	5
pN – stage	0	21	20.8
	1	22	21.8
	2	27	26.7
	3a	18	17.8
	3b	13	12.9
Overall stage	Ib	14	13.9
	IIa	22	21.8
	IIb	20	19.8
	IIIa	25	24.7
	IIIb	17	16.8
	IIIc	3	3
Pathohistological tumour grade	1	5	5
	2	29	28.7
	3	65	64.3
	unknown	2	2
Borrman type	1	0	0
	2	11	10.9
	3	30	29.7
	4	17	16.8
	unknown	43	42.6
Growth type according to Lauren	diffuse	28	27.7
	intestinal	45	44.6
	mixed	19	18.8
	unknown	9	8.9
Perineural invasion	yes	49	48.5
	no	36	35.7
	unknown	16	15.8
Lymphovascular invasion	yes	48	47.6
	no	16	15.8
	unknown	37	36.6
Angioinvasion	yes	21	20.8
	no	52	51.5
	unknown	28	27.7
HP	yes	9	8.9
	No	44	43.6
	unknown	48	47.5

pT = pathological T-stage; pN = pathological N-stage; HP = Helicobacter pylori infection

**TABLE 2.** Toxicity of adjuvant radiochemotherapy

**Toxicity**	**NCI grade (%)**					

	**0**	**1**	**2**	**3**	**4**	**Total**

**Nausea, vomiting**	**56.4**	**34.6**	**4**	**5**	**0**	**100**
Stomatitis	90.1	7.9	1	1	0	100
Diarrhoea	86.1	10.9	2	1	0	100
Hand-foot syndrome	73.3	10.9	6.9	8.9	0	100
Dysphagia	73.3	25.7	1	0	0	100
Acute coronary syndrome	96	4	0	0	0	100
Alopecia	97	3	0	0	0	100
Infection	43.6	8.9	28.7	17.8	1	100
Leucocyte count	25.8	36.6	30.7	5.9	1	100
Haemoglobin level	28.7	62.4	8.9	0	0	100
Platelet count	52.4	42.6	4.	1	0	100

**TABLE 3.** Multivariate analysis of survival

**Prognostic factors**	**Locoregional control**			**Disease-free survival**			**Disease-specific survival**			**Overall survival**		

	**HR**	**95% CI**	**P-value**	**HR**	**95% CI**	**P-value**	**HR**	**95% CI**	**P-value**	**HR**	**95% CI**	**P-value**
pN- stage: - 0+1+2 - 3	17.97	1.32–244.69	0.03	7.38	2.93–18.59	<0.0001	9.67	3.54–26.36	<0.0001	7.40	3.08–17.78	<0.0001
Perineural invasion: - Yes - No	0.57	0.07–4.90	NS	2.89	1.07–7.77	0.036	3.45	1.16–10.27	0.026	2.55	1.08–6.02	0.032
Splenectomy: - Yes - No	22.42	1.20–417.44	0.037	3.07	1.12–8.48	0.03	4.37	1.50–12.71	0.007	5.59	2.27–13.81	<0.0001
Treatment according to the protocol: - Yes - No	0.19	0.02–1.88	NS	0.30	0.11–0.80	0.016	0.27	0.10–0.78	0.015	0.28	0.12–0.66	0.004

HR = hazard ratio; CI = confidence interval; NS = not significant

## References

[1] (2013). Cancer in Slovenia 2009.

[2] Buyukasik O, Hasdemir AO, Gulnerman Y, Col C, Ikiz O (2010). Second primary cancers in patients with gastric cancer. Radiol Oncol.

[3] Rajev L (2010). Treatment options for surgically resectable gastric cancer. Curr Treat Options Oncol.

[4] Buergy D, Lohn F, Baack T, Siebenlist K, Haneder S, Michaely H (2012). Radiotherapy for tumors of the stomach and gastoesophageal junction - a review of its role in multimodal therapy. Radiat Oncol.

[5] Macdonald JS, Smalley SR, Benedetti J, Hundahl SA, Estes NC, Stemmermann GN (2001). Chemoradiotherapy after surgery compared with surgery alone for adenocarcinoma of the stomach or gastroesophageal junction. N Engl J Med.

[6] Oblak I, Velenik V, Anderluh F, Strojan P (2007). Results of adjuvant radiochemotherapy for gastric adenocarcinoma in Slovenia. Eur J Surg Oncol.

[7] Oblak I, Anderluh F, Velenik V (2009). Postoperative radiochemotherapy for gastric adenocarcinoma: long term results. Radiol Oncol.

[8] Rossi D, Alessandroni P, Catalano V, Giordani P, Fedeli A, Baldelli AM (2007). Safety profile and activity of lower capecitabine dose in patients with meta-static breast cancer. Clin Breast Cancer.

[9] Walko CM, Lindley C (2005). Capecitabine: a review. Clin Ther.

[10] Ocvirk J, Rebersek M, Skof E, Hlebanja Z, Boc M (2012). Randomized prospective phase II study to compare the combination chemotherapy regiment epirubicin, cisplatin and 5-fluorouracil with epirubicin, cisplatin and capecitabine in patients with advanced or metastatic gastric cancer. Am J Clin Oncol.

[11] Twelves C, Scheithauer W, McKendrick J, Seitz JF, Van Hazel G, Wrong A (2012). Capecitabine versus 5-fluorouracil/folinic acid as adjuvant therapy for stage III colon cancer: final results from the X-ACT trial with analysis by age and preliminary evidence of a pharmacodynamic marker of efficacy. Ann Oncol.

[12] American Joint Committee on Cancer (2009). AJCC cancer staging manual.

[13] Ajani JA, Welch SR, Raber MN, Fiels WS, Krakoff IM (1990). Comprehensive criteria for assessing therapy-induced toxicity. Cancer Invest.

[14] Kaplan EL, Meier P (1958). Nonparametric estimation from incomplete observations. J Am Stat Assoc.

[15] Peto R, Pike MC, Armitage P, Breslow NE, Cox DR, Howard SV (1977). Design and analysis of randomized clinical trials requiring prolonged observation of each patient. II. Analysis and examples. Br J Cancer.

[16] Cox DR (1972). Regression models and life-tables. J R Stat Soc Bull.

[17] Greenlee RT, Murray T, Bolden S, Wingo PA (2000). Cancer statistics 2000. Ca Cancer J Clin.

[18] Ajani JA (1998). Chemotherapy for gastric carcinoma: new and old options. Oncology (Huntingt).

[19] Gunderson LL (2002). Gastric cancer: patterns of relapse after surgical resection. Semin Radiat Oncol.

[20] Park SH, Kim DY, Heo JS, Lim DH, Park CK, Lee KW (2003). Postoperative chemotherapy for gastric cancer. Ann Oncol.

[21] Kim S, Lim DH, Lee J, Kang WK, McDonald JS, Park CH (2005). An observation study suggesting clinical benefit for adjuvant postoperative chemoradiation in a population of over 500 cases after gastric resection with D2 nodal dissection for adenocarcinoma of the stomach. Int J Radiat Oncol Biol Phys.

[22] Smalley SR, Benedetti JK, Haller DG, Hundahl SA, Estes NC, Ajani Ja (2012). Updated analysis of SWOG-directed intergroup study 0116: a phase III trial of adjuvant radiochemotherapy versus observation after curative gastric cancer resection. J Clin Oncol.

[23] Dikken JL, Jansen EP, Cats A, Bakker B, Hartgrink HH, Kranenbarg EM (2010). Impact of the extent of surgery and postoperative chemoradiotherapy on recurrence patterns in gastric cancer. J Clin Oncol.

[24] Lee J, Lim do H, Kim S, Park SH, Park JO, Park YS (2012). Phase III trial comparing capecitabine plus cisplatin versus capecitabine plus cisplatin with concurrent capecitabine radiotherapy in completely resected gastric cancer with D2 lymph node dissection: the ARTIST trial. J Clin Oncol.

[25] Zhu WG, Xua DF, Pu J, Zong CD, Li T, Tao GZ (2012). A randomized, controlled, multicenter study comparing intensity-modulated radiotherapy plus concurrent chemotherapy with chemotherapy alone in gastric cancer patients with D2 resection. Radiother Oncol.

[26] Yu C, Yu R, Zhu W, Song Y, Li T (2012). Intensity-modulated radiotherapy combined with chemotherapy for the treatment of gastric cancer patients after standard D1/D2 surgery. J Cancer Res Clin Oncol.

[27] Jansen EP, Boot H, Saunders MP, Crosby TD, Dubbelman R, Bartelink H (2007). A phase I-II study of postoperative capecitabine-based chemoradiotherapy in gastric cancer. Int J Radiat Oncol Biol Phys.

[28] Kim JP, Lee JH, Kim SJ, Yu HJ, Yang HK (1998). Clinicopathologic characteristics and prognostic factors in 10783 patients with gastric cancer. Gastric Cancer.

[29] Orditura M, De Vita F, Muto P, Vitiello F, Murino P, Lieto E (2010). Adjuvant chemotherapy in patients with stage III or IV radically resected gastric cancer. Arch Surg.

[30] Yao JC, Mansfield PF, Pisters PWT, Feig BW, Janjan NA, Crane C (2003). Combined modality therapy for gastric cancer. Semin Surg Oncol.

[31] Avital I, Pisters PWT, Kelsen DP, Willett CG, DeVita VT, Lawrence TS, Rosenberg SA (2004). Cancer of the stomach. Cancer; principles & practice of oncology.

[32] Willett CG, Gunderson LL, Halperin EC, Perez CA, Brady LW (2008). Stomach. Principles and practice of radiation oncology.

[33] Chua YJ, Cunningham D (2007). The UK NCRI MAGIC trial of perioperative chemotherapy in resectable gastric cancer: implications for clinical practice. Ann Surg Oncol.

[34] Ikeguchi M, Kaibara N (2004). Lymph node metastasis at the splenic hilum in proximal gastric cancer. Am Surg.

[35] Huang CM, Wang JB, Lu HS, Zheng CH, Li P, Wie JW (2009). Prognostic impact of splenectomy on advanced proximal gastric cancer with No.10 lymph node metastasis. Chin Medl J.

